# Local Therapy of the Primary Tumor: An Option for Metastatic Urothelial Carcinoma in the Era of Novel Combination Therapies?

**DOI:** 10.1016/j.euros.2025.05.016

**Published:** 2025-07-14

**Authors:** Renate Pichler, José Daniel Subiela, Roman Mayr, Marco Moschini, Roger Li, Benjamin Pradére

**Affiliations:** a Department of Urology, Comprehensive Cancer Center Innsbruck, Medical University of Innsbruck, Innsbruck, Austria; b Department of Urology, Instituto Ramón y Cajal de Investigación Sanitaria, Hospital Universitario Ramón y Cajal, Universidad de Alcalá, Madrid, Spain; c Department of Urology, Caritas St. Josef Hospital, University of Regensburg, Regensburg, Germany; d Division of Experimental Oncology/Unit of Urology, Urological Research Institute, IRCCS Ospedale San Raffaele, Milan, Italy; e Vita-Salute San Raffaele University, Milan, Italy; f Department of GU Oncology, H. Lee Moffitt Cancer Center and Research Institute, Tampa, FL, USA; g Department of Urology UROSUD, La Croix Du Sud Hospital, Quint-Fonsegrives, France

**Fig. 1:**
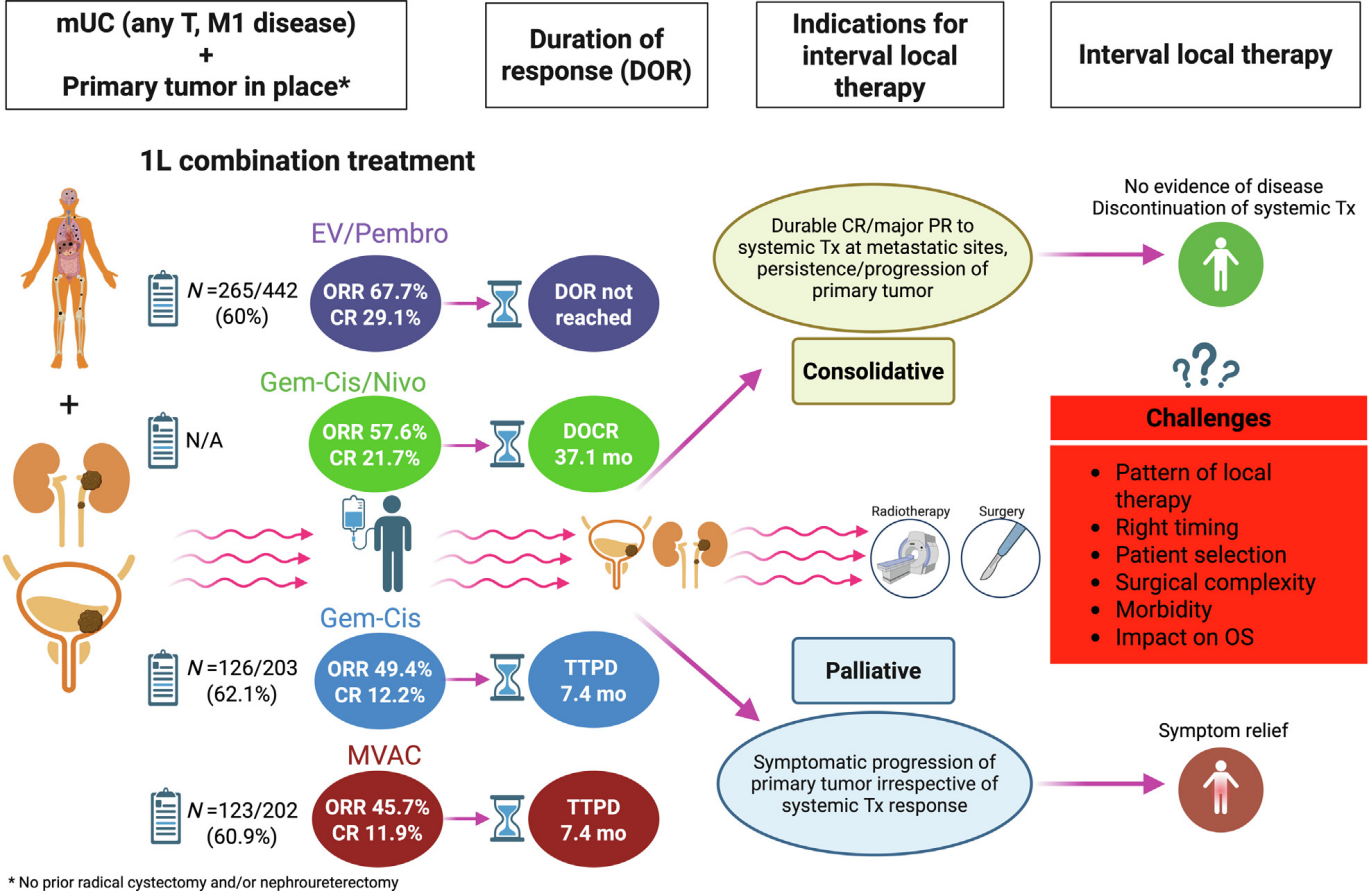
Schematic overview of clinical scenarios for interval local therapy of the primary tumor in place in metastatic urothelial carcinoma (mUC). More than 60% of mUC patients received systemic therapy while retaining their primary tumor. In the EV-302 study, 265 of 442 patients (60%) in the EV/P treatment arm had not undergone prior radical cystectomy and/or nephroureterectomy [5]. Similarly, 60.9% to 62.1% of patients treated with cisplatin-based chemotherapy (GC or MVAC) had not received prior definitive surgery of the primary tumor [1]. In the era of novel combination therapies, two distinct clinical indications for deferred local therapy of the primary tumor might be proposed. First, palliative local therapy is an option in cases where persistent symptoms caused by the primary tumor cannot be adequately controlled with less invasive methods. Second, consolidative local therapy may be considered when there is a durable CR of metastatic lesions but persistence—or even isolated progression—of the primary tumor, potentially offering a therapeutic benefit.

Patients with metastatic urothelial carcinoma (mUC) have historically poor prognoses, with estimated 5-year survival rates of 5–15% [1]. In the first-line setting, cisplatin-based chemotherapy using gemcitabine/cisplatin (GC) or methotrexate, vinblastine, doxorubicin, and cisplatin (MVAC) achieved a median overall survival (OS) of up to 15 months, with complete response (CR) rates of 12% over a median duration of approximately 7 months [1]. Retrospective studies have suggested a survival benefit from combining chemotherapy with additional local therapy (targeting the primary tumor and metastatic sites) in oligometastatic UC [2,3]. A recent Delphi consensus paper also recommends local metastases-directed therapy in case of favourable response of oligometastatic disease to systemic therapy [4]. Focusing on the primary tumor, deferred local therapy is often performed for palliative reasons, particularly when symptoms control (e.g., hematuria, hydronephrosis, urinary obstruction, dysuria, pain) could not be achieved through less invasive methods, irrespective of the systemic therapy response at metastatic lesions. Different clinical scenarios of deferred local therapy for the primary tumor in mUC are summarized in Fig. 1**.**

Two novel first-line combination therapies, enfortumab vedotin plus pembrolizumab (EV/Pembro) and GC plus nivolumab (GC/Nivo), have recently emerged as new standards in the treatment of first line metastatic UC [5,6]. These regimens have nearly doubled the median OS compared to cisplatin-based chemotherapy, extending median OS to approximately 32 months and achieving durable CR rates of up to 30% [5]. The median duration of CR in patients receiving GC/Nivo was 37.1 months [6], and the median duration of response using EV/Pembro is still not reached [5]. A prognostically favorable subgroup in terms of therapy response includes patients with lymph node-only disease. In this cohort, objective response rates (ORR) ranged from 77.5% in the EV-302 study [5] to 81.5% in the CheckMate 901 [6], with striking CR rates of 63% in patients treated with GC/Nivo [6].

Given these promising results, the question arises whether interval local consolidative therapy of the primary tumor might play any role in patients with sustained CR to EV/Pembro or GC/Nivo at metastatic sites to achieve complete remission, potentially leading to eradication of disease and enabling the discontinuation of further systemic treatment. In metastatic renal cell carcinoma (mRCC), patients achieving a CR or a significant partial response (>80%) to first line immune checkpoint inhibitor (ICI) therapy at metastatic lesions are increasingly considered for deferred cytoreductive nephrectomy [7].

While up to 70% of mRCC patients in pivotal ICI combination therapy trials underwent primary cytoreductive nephrectomy [8], approximately 60% of metastatic UC patients were treated with their primary tumor in place (i.e., no prior cystectomy or nephroureterectomy) [1,5]. This discrepancy raises several important questions regarding the optimal clinical management of the primary tumor, which remain unanswered by both pivotal trials [5,6].

First, how does the primary tumor in place respond to EV/Pembro or GC/Nivo? In the first retrospective study by Roberson *et al* [9], a pathological CR (ypT0 N0) of primary tumors was observed in 43% following EV and/or ICI systemic combination therapy, with a pathological downstaging in 82% [9].

Secondly, is there any concordance between treatment response in the intact primary UC (bladder and/or upper urinary tract) and metastatic lesions during EV/Pembro or GC/Nivo therapy? This is a crucial consideration when deciding on potential interval local therapy in clinical practice. Interestingly, among the 23 patients who experienced pathological downstaging at the time of surgery, 61% achieved a CR at metastatic sites following EV and/or ICI combination therapy [9].

A critical consideration is the appropriate course of action in case the primary tumor does not respond to systemic therapy. Since the main rationale for interval local therapy (radiotherapy or surgery) is due to discrepant responses at the primary and metastatic sites (i.e. a favorable response at metastatic sites, but with persistent or progressive/symptomatic disease in the primary tumor), this factor must be accounted for when assessing the impact of deferred local consolidative therapy on OS.

Another key question is whether interval surgery of the primary tumor, following ICI-based combination therapy, influences surgical complexity in metastatic disease. Although there are no data on this in the metastatic setting of both pivotal trials [5,6] yet, Roberson et al. confirmed a similar rate of high-grade surgical complications (Clavien-Dindo Grade III–V: 18%) following consolidative surgery, comparable to rates observed with contemporary surgery for localized disease [9]. Furthermore, studies in the neoadjuvant setting show that prior immunotherapy does not have a negative impact on the surgical complexity of radical cystectomy. In detail, the addition of durvalumab to chemotherapy in the NIAGARA trial did not impact the rate or timing of radical cystectomy, and did not increase the rate of surgical complications by Clavien-Dindo Classification (Grade III-V: 16% GC/Durvalumab vs. 16% GC) [10]. In the SunRISe-4 study, the combination of intravesical TAR-200 + cetrelimab had no negative effect on surgery, thus not delaying radical cystectomy [11].

With the advent of novel ICI-based combination therapies in the first-line setting, which significantly prolonged OS and achieved high and durable CR rates in metastatic UC, we anticipate that local therapy of the primary tumor may increasingly shift beyond a purely palliative indication to an approach aimed at achieving no evidence of disease in the near future. This might be particularly relevant for patients who achieve a high and durable response to systemic therapy at metastatic sites (CR or major PR), with the possible goal of enabling systemic therapy discontinuation and thus reducing treatment-related toxicity.

However, key challenges include determining the optimal timing for deferred local therapy, selecting the most appropriate modality (surgery vs. radiotherapy), identifying novel biomarkers to select subgroups most likely to benefit (e.g., lymph node-only disease, oligometastatic disease), assessing the impact of interval local therapy on survival, and evaluating the potential for increased surgical complexity following prior ICI therapy in metastatic UC. Future trials as well as more real-world data are needed to investigate the impact of interval local therapy on survival after ICI-based combination treatments in metastatic UC patients with an intact primary tumor. Hopefully, both pivotal studies [5,6] can shed more light in the tunnel through further analyses after a longer follow-up period.

  ***Conflicts of interest*:** The authors have nothing to disclose.
